# GATA6 in pancreatic cancer initiation and progression

**DOI:** 10.1016/j.gendis.2024.101353

**Published:** 2024-06-17

**Authors:** Muyuan Ma, Jianhong An, Tingting Jiang, Keping Xie

**Affiliations:** Center for Pancreatic Cancer Research, The South China University of Technology School of Medicine, Guangzhou, Guangdong 510006, China

**Keywords:** GATA6, Inflammation, Molecular diagnosis, Pancreas, Targeted therapy, Tumorigenesis

## Abstract

Pancreatic ductal adenocarcinoma (PDA) is a lethal malignancy characterized by insidious onset and lack of effective therapy. The molecular pathogenesis of PDA remains to be understood fully. Transcriptional factor GATA6 is an important transcriptional regulator in normal pancreas development, particularly in the initial specification and differentiation of the pancreas. Recent studies have linked pancreatic malignancy closely to GATA6. Increased levels of GATA6 expression enhance pancreatic cancer cell growth. GATA6 emerges as a lineage-specific oncogenic factor in PDA, augmenting the oncogenic phenotypes of PDA cells upon its overexpression. However, elevated GATA6 levels are correlated with well-differentiated tumors and a more favorable patient prognosis. Experimental evidence in genetic mouse models has revealed a tumor-suppressive role for GATA6. The circumstantial roles of GATA6 in pancreatic tumorigenesis remain to be defined. This review aims to elucidate recent advances in comprehending GATA6, emphasizing its crucial roles in both pancreas physiology and pathology. Special attention will be given to its involvement in PDA pathogenesis, exploring its potential as a novel biomarker and a promising therapeutic target for PDA.

## Instruction

Pancreatic cancer could originate from both the exocrine and endocrine pancreas. Pancreatic ductal adenocarcinoma (PDA) accounts for over 90% of exocrine pancreatic cancer cases, with variants being uncommon and uninformative for management decisions.^1^ Adenosquamous carcinoma represents a lesser-acknowledged and infrequent form of PDA, showcasing characteristics of both adenocarcinoma and squamous cell carcinoma, along with a blend of glandular and squamous differentiation.^2^ The exocrine pancreas also produces other carcinomas with acinar differentiation, *e.g.*, acinar cell carcinomas, pancreatoblastomas, and carcinomas with mixed histology. These uncommon carcinomas are associated with an unfavorable prognosis and their therapeutic options remain to be defined. The next common subtypes of pancreatic cancer following PDA are those of neuroendocrine origin and are described as pancreatic neuroendocrine tumors or islet cell tumors of the pancreas. Pancreatic neuroendocrine tumors usually arise sporadically and the majority of them are indolent, in which surgical and locoregional therapy are preferred.^3^

Being the prevalent malignancy of the pancreas, PDA presents an exceedingly bleak prognosis and constitutes a significant medical challenge. PDA accounts for approximately 7% of total cancer-related mortalities, with a 5-year survival rate hovering around 5%.^4^^,^^5^ PDA harbors mutational activation in *KRAS* and inactivation of *CDKN2A*, *TP53*, and *SMAD4*.^6^, ^7^, ^8^ Although the molecular basis of PDA is well investigated, the management of PDA remains a huge clinical challenge, including early identification and diagnosis, anticipation of therapeutic responsiveness, and forecasting of outcomes. The significance of using common two biomarkers, carcinoembryonic antigen (CEA) and carbohydrate antigen 19-9 (CA19-9) is limited in PDA management.^9^, ^10^, ^11^, ^12^ This review aims to elucidate recent advances in comprehending GATA-binding protein 6 (GATA6), emphasizing its crucial roles in both pancreas physiology and pathology. Special attention will be given to its involvement in PDA pathogenesis, exploring its potential as a novel biomarker and a promising therapeutic target for PDA.

## Pancreatic cancer molecular classification

The molecular classification of PDA can be founded on either individual genetic marker categorization, arrangements of genomic anomalies, transcriptomic profiles, or alternative approaches^13^^,^^14^ Among these categorizations, the transcriptomic subtyping methodologies within pancreatic cancer draw from tactics employed in classifying other neoplasms.^15^^,^^16^ Transcriptomic assessment based on the epithelial component of PDA enables the categorization into distinct phenotypic subtypes referred to as classical and basal-like (*i.e.*, Moffitt subtype). The classical subtype, marked by more frequent suitability for resection, exhibits heightened differentiation levels frequently correlated with fibrosis and inflammation. Conversely, the basal-like subtype is linked with an inferior clinical prognosis and diminished differentiation status.^17^^,^^18^ Molecular categorization is closely associated with histological attributes. Hayashi et al conducted an integrated analysis encompassing multiple regions, histology, expression profiles, and genetic changes. Their findings indicated a correlation between squamous histological morphology and the basal-like subtype, whereas glandular morphology was associated with the classical subtype. Noteworthy is their deduction that regions exhibiting squamous characteristics represent a subclonal population within a glandular tumor.^19^ Categorizing PDA more effectively enhances personalized patient care strategies and/or risk assessment (such as primary surgery versus neoadjuvant therapy), refines the selection of systemic therapeutic regimens, encompassing enrollment in clinical trials (prediction of response), and fosters enhanced organization of research and therapeutic advancement endeavors.

Innovative transcriptomic subclasses are also reported.^14^ In 2011, Collisson et al established three categories: classical, quasi-mesenchymal (QM-PDA), and exocrine-like. The QM-PDA category demonstrated an association with elevated tumor grade and unfavorable survival outcomes, while the classical subtype exhibited the presence of the endodermal lineage-specifying transcription factor GATA6 and showcased dependency on KRAS.^20^ In 2016, Bailey et al conducted an mRNA hybridization analysis, delineating four subtypes, namely squamous, pancreatic progenitor, immunogenic, and aberrantly differentiated endocrine exocrine (ADEX), which exhibit concordance with histopathological attributes. The squamous subtype demonstrates hypermethylation and simultaneous down-regulation of genes governing the determination of pancreatic endodermal cell fate (such as GATA6), resulting in the complete loss of endodermal identity and an unfavorable prognosis. Pancreatic progenitor tumors manifest preferential expression of genes related to early pancreatic development. ADEX tumors exhibit up-regulation of genes overseeing networks associated with KRAS activation, exocrine, and endocrine differentiation. Immunogenic tumors feature elevated immune networks, including pathways linked to acquired immune suppression.^21^ From these findings, it can be deduced that there exist variances in the molecular progression of distinct pancreatic cancer subtypes, which consequently reveal prospects for the advancement of therapeutic interventions. Collisson et al proposed a consolidated terminology that encompasses two broad subtypes, namely squamous and classical–pancreatic, encompassing the classical–progenitor and ADEX (possibly nested within exocrine-like) subtypes within the latter. The classical–progenitor classification further divides into the immunogenic progenitor and pure classical progenitor subcategories.^14^ However, even these well-established categorizations could prove insufficient and fall short of effectively depicting the heterogeneity of PDA, particularly when tumors concurrently encompass a multitude of cellular phenotypes.

Stromal subtypes have been categorized into two distinct groups, normal and activated.^18^ Presently, there is no apparent direct correlation between these stromal subtypes and epithelial subtypes. Puleo et al propose the potential distinctness of these stromal subtypes as separate subcategories, yet the uncertainty remains regarding whether these subtypes constitute orthogonal parameters for the classical–pancreatic and squamous subtypes.^22^ Significantly, the phenotypic demarcation is not impeccably precise, and certain instances of PDA reveal the coexistence of distinct cancerous cell lineages within a singular tumor. This observation underscores the notion that the tumor mass in PDA exhibits marked heterogeneity, encompassing a variety of malignant and stromal cell classifications.^23^ Peng et al reported that the malignant ductal subtype exhibited a discernible gene expression profile, characterized by pronounced proliferation and migratory subgroups. They suggested that these subgroups of cells align with basal-like cell characteristics, constituting about 6.30% of their sample's cellular composition. In contrast, the classical subtype constitutes 26.95% of the cellular population.^23^

Dijk et al conducted an exploration of gene expression through analysis and unsupervised categorization on a meticulously annotated RNA sequencing expression dataset derived solely from PDA, within a single research institution. Their study revealed the presence of four discrete PDA subgroups, which exhibited correlations with distinctive clinical presentations. Their identified subtype demonstrated concurrence with previously reported subtypes in terms of sample identification. However, the biological characteristics of the compound subtype-specific to the pancreas exhibited clear parallels with the mesenchymal subtype. They propose that the compound subtype emerges as a consequence of intra-tumor heterogeneity.^24^

In an alternative study, single-cell RNA sequencing analysis was conducted on six organoids representative of the classical subtype of PDA. This endeavor led to the recognition of four predominant cellular clusters, each characterized by a distinct gene expression pattern linked to specific biological traits and molecular indicators. Despite the preliminary classification of these tumors as classical, one cluster consistently observed across all patients exhibited a basal-like phenotype based on bulk RNA sequencing.^25^ These findings illustrate the unanticipated extent of heterogeneity in pancreatic malignancies, underscoring that basal-like cells, bearing a notably aggressive phenotype, are more prevalent than anticipated. Elucidating this within-tumor heterogeneity holds pivotal significance in comprehending the evolution of PDA and in conceptualizing novel perspectives that could advance tailored and efficacious therapeutic approaches.

## General structure and function of GATA6

**Figure 1:**
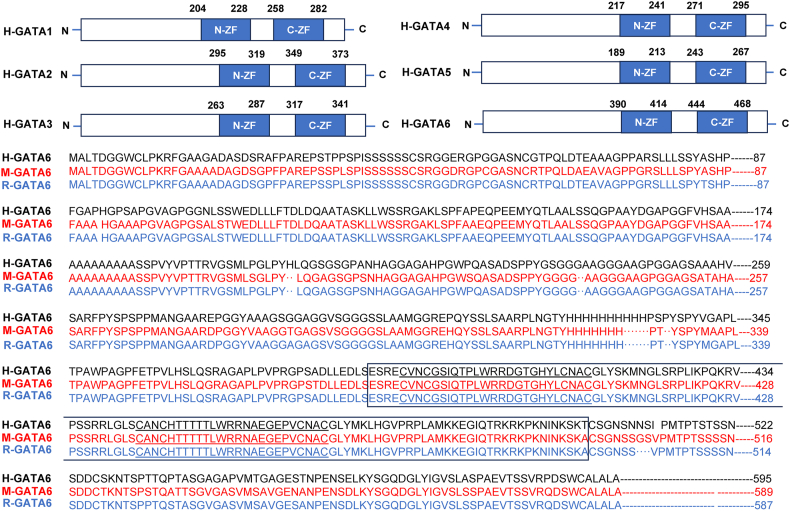
The GATA proteins with different Zinc finger domains, and the sequence comparison of GATA6 from different species. The full length of GATA1, 2, 3, 4, 5, and 6 contains the two Zinc finger domains (N-ZF and C-ZF). The sequences of GATA6 protein from human, mouse and rat are aligned together. The Zinc finger region is boxed and the N-finger and C-finger are underlined. N-ZF, N-Zinc finger domain; C-ZF, C- Zinc finger domain; H, human; M, mouse; R, rat.

GATA6 is a member of the GATA family of zinc-finger transcriptional regulators, named after the conserved base sequence (G/A) GATA(A/T). A diagram of GATA6 with different zinc finger domains, along with other GATA proteins is compared in Figure 1. GATA6 features conserved tandem zinc fingers with the structure (CVNC-X17-CNAC)-X29-(CXNC-X17-CNAC) and plays an essential role in coordinating the development and precise gene regulation of diverse tissues, including the heart and gastrointestinal tract. Recognizing the sequence (A/T/C) GAT(A/T) (A), GATA6 interacts with other transcriptional regulators through its zinc-finger domain. The mRNA of GATA6 utilizes two initiation codons in tandem for translation, giving rise to both L- and S-type GATA6 isoforms through permissive ribosome scanning. Notably, GATA6 is susceptible to cAMP-dependent proteolysis facilitated by the proteasome in a heterologous expression system. These protein-centric characteristics of GATA6 contribute to identifying target genes and aid in delineating GATA6's *in vivo* binding sites, thereby enhancing our understanding of the intricate network of gene regulation modulated by GATA6.^26^ Meanwhile, GATA6 can regulate the activity of signaling pathways and influence the development, differentiation, and carcinogenesis of the pancreas, lungs, intestines, and other organs (Table 1).

**Table 1:** GATA6 associated pathways in regulating development and differentiation, and carcinogenesis.

Pathways associated with GATA6	Correlated factors	Influence	Effects on the pathway	Experimental model	Reference
Hedgehog	Shh	GATA6 regulates pancreatic endoderm specification during patterning of the gut tube.	GATA6 suppresses the activity of the Shh.	Mouse models; Cells	PMID:26932670
	Shh	GATA6 in the limb bud inhibits hindlimb polydactyly.	GATA6 inhibits the expression of Shh.	Mouse models	PMID:24415953
	Shh	GATA6 suppresses the proliferation, migration of lung squamous cell carcinoma cells.	GATA6 transcriptionally suppresses Shh expression.	Cells	PMID:31442607
	Ihh	GATA6 affects the differentiation of Mouse F9 cells into primitive extraembryonic endoderm.	GATA6 activates Hedgehog signaling during primitive extraembryonic endoderm formation.	cells	PMID:29119099
Notch	Dll1	GATA6 affects the proliferation of the goblet cells through Notch signaling.	GATA6 deletion results in alterations in Notch signaling in ileum.	Mouse models	PMID:21262227
	Dll1	GATA6 play crucial role in the maintenance of the structure and differentiation for intestine epithelium.	GATA6 and GATA4 enhance Dll1 expression.	Mouse models	PMID:24929016
	JAG2	GATA6 affects cancer cell growth, migration, invasion, and metastasis through BMP4.	GATA6 stimulate Bmp4 transcription, and BMP4 up-regulates JAG2.	Mouse models	PMID:26395571
TGF-β	TGFβ1/2	play critical role for the survival and normal function of endothelial cells.	GATA6 suppresses the expression of TGFβ1 and TGFβ2.	Cells	PMID:21127043
	Smad2	GATA6 inhibits mesendodermal induction by Smad2 signaling.	GATA6 interacts with Smad2 and inhibits transcription activity of Smad2.	Zebrafish models	PMID:21669877
	Sox17α and HNF1β	GATA6 is the dominant GATA factor in the maintenance of endodermal gene expression by TGFβ signal pathway in the gastrulating embryos.	GATA6 is a direct activator of early endodermal genes Sox17α and HNF1β.	Xenopus models	PMID:15659482
VEGFR	VEGFR	GATA6 regulates the lymphatic dissemination of bladder cancer.	GATA6 inhibits the transcription of VEGF factor family VEGF-C.	Cells	PMID:19501129
Wnt	β-catenin	GATA6 are required for the maintenance of normal function and number of goblet-like cells and Paneth cells.	GATA6 deletion changes the expression of crypt Wnt targets in ileum.	Mouse models	PMID:21262227
	Wnt6	GATA6 affects the differentiation of Mouse F9 cells.	GATA6 directly activates Wnt6.	Cells	PMID:29119099
	DKK1	GATA6 promotes pancreatic carcinogenesis.	GATA6 activates the canonical WNT signaling.	cells	PMID:21811562
	p300	A p300/GATA6 axis determines the differentiation activity of Wnt signaling, and affect pancreatic cancer cells resistant to Wnt inhibition.	p300/GATA6 axis changes Wnt dependency of pancreatic cancer and its subtype.	Mouse models; Cells	PMID:35536676
	β-catenin	GATA6 activates fibroblast, and promotes tracheal fibrosis via the Wnt/β-catenin signaling.	GATA6 deficiency causes downregulation of GSK3β-dependent phosphorylation and the degradation of β-catenin.	Rat models; Cells	PMID:36682592

Note: GATA6 can affect Hedgehog pathway by regulating the activity of Shh and Ihh; Notch pathway by regulating Dll1 and JAG2; TGF-β pathway by regulating TGFβ1/2, Smad2, Sox17α and HNF1β; VEGFR pathway by regulating VEGFR; and Wnt pathway by regulating β-catenin, Wnt6, DKK1 and p300.

## GATA6 in pancreas development

Functioning as a factor specific to lineages, a chromatin remodeling entity, a pluripotency determinant, and a pioneering influencer, GATA6 plays integral roles across diverse phases of pancreas development. Numerous inactivating mutant alleles of GATA6 have been associated with the occurrence of pancreatic agenesis in humans.^27^

The GATA gene families hold essential positions in governing cellular destiny determination, the process of proliferation, migration dynamics, and the intricate orchestration of organogenesis within organs originating from both endoderm and mesoderm lineages in vertebrates. GATA1 and GATA2 play pivotal roles in the hematopoietic system.^28^^,^^29^ GATA1 is significantly involved in the differentiation of red blood cells and platelets, while GATA2 holds a critical function in regulating and differentiating hematopoietic stem cells. GATA3 assumes a significant role within the immune system, particularly in the differentiation of T cells. Its regulation is crucial for the differentiation of Th2 cells, contributing to the modulation of allergic responses and immune reactions.^30^ GATA4, GATA5, and GATA6 constitute the secondary subgroup, holding significance in the differentiation of tissues originating from both endoderm and mesoderm lineages.^31^^,^^32^ GATA5 predominantly presents its expression within the evolving heart, the pulmonary mesenchyme, and specific smooth muscle cells within distinct tissues.^33^ Solely GATA6 and GATA4 play critical roles in pancreas development, serving as pioneering factors in initiating the expression of tissue-specific genes. These factors sustain their expression throughout the development of both the dorsal and ventral pancreatic bud epithelia. However, with the advancement of pancreatic development, GATA6 becomes restricted to the endocrine and ductal compartments within the typical adult pancreas,^34^ whereas GATA4 continues to exhibit significant expression in the acinar tissue.^35^^,^^36^

In murine pancreas development, GATA6 maintains a close association with GATA4, and there exists a certain level of redundancy between mouse GATA4 and GATA6 in the regulation of pancreas development. Xuan et al engineered pancreas-specific deletions of GATA4 and GATA6, noting that the absence of either GATA4 or GATA6 in the pancreas resulted in mild pancreatic defects, which spontaneously resolved during the postnatal period. However, the concurrent elimination of both GATA4 and GATA6 in the pancreas led to severe pancreatic agenesis. This outcome was attributed to disruptions in the proliferation of pancreatic progenitor cells, abnormalities in the formation of branched structures, and subsequent failure to initiate the differentiation of progenitor cells expressing carboxypeptidase A1 (CPA1) and neurogenin 3 (NEUROG3). Despite GATA4 and GATA6 being expressed early across the pre-pancreatic endoderm, the specification of the pancreas remained unaffected in the double knockout embryos. Therefore, the data put forward the notion that the role of GATA factors is dispensable in triggering the pancreatic developmental process within the foregut endoderm. Elucidation of the double knockout pancreas on a molecular level implies that GATA function becomes necessary after the specification of the pancreatic multipotent progenitor population (marked by Pdx1, Ptf1a, and Sox9 expression), occurring before the subsequent establishment of lineage-specific endocrine (Neurog3^+^) and exocrine (Cpa1^+^) precursor populations. Among all analyzed double knockout embryos, the deficiencies in pancreatic development manifested earlier and exhibited heightened severity within the dorsal pancreas in comparison to the ventral pancreas. This observation implies that the GATA factors hold a more pivotal role during the early phases of dorsal pancreas development, potentially due to their established involvement in retinoic acid signaling mediation. Specifically, retinaldehyde dehydrogenase 2 (RALDH2; Aldh1a2), the primary enzyme responsible for embryonic retinoic acid production, notably governs the development of the dorsal pancreas. In the ventral pancreas, it is conceivable that the GATA factors could facilitate pancreas differentiation in response to an alternate signaling pathway during a subsequent developmental stage.^37^ Carrasco et al employed conditional inactivation to target GATA4 and GATA6 specifically within the pancreas. Singular inactivation of either gene showcased minimal influence on pancreas formation, indicating functional redundancy. However, the simultaneous absence of GATA4 and GATA6 in double mutant mice resulted in the absence of pancreas development, leading to postnatal demise and the onset of hyperglycemia. Distinct anomalies in the morphology of GATA4/GATA6 mutant pancreas became evident during embryonic growth, stemming from impaired cell proliferation and differentiation, which hindered epithelial expansion. In the mutant pancreatic epithelium, there was a significant reduction in the population of multipotent pancreatic progenitors, including PDX1^+^ cells. Interestingly, the removal of a single GATA6 allele in the context of a GATA4 conditional knockout markedly reduced pancreatic mass. Conversely, the presence of a single wild-type GATA4 allele in GATA6 conditional knockout mice proved adequate for normal pancreatic development, indicating diverse contributions of GATA factors to pancreas formation.^38^

These studies emphasize the indispensable and redundant functions of GATA4 and GATA6 in the development and differentiation of the pancreas, highlighting both conserved and non-conserved roles of these factors in both murine and human pancreas.

Interestingly, in human cases, the absence of one functional copy of individual GATA factors led to pancreatic agenesis. In contrast, mice harboring homozygous pancreas-specific deletions of distinct GATA factors exhibited only minor cellular anomalies and maintained physiological normalcy throughout their lifespan. Pancreatic significant developmental anomalies are observed only when all alleles of both GATA4 and GATA6 are deleted. The variation in observed phenotypes might arise from the inherent characteristics of the GATA mutations. Individuals harboring mutations in GATA6 exhibit compromised GATA6 activity across all tissues, encompassing nonpancreatic tissues that might impart instructive cues necessary for the initiation of proper pancreas formation and development. Moreover, the evidence from human data implies a greater susceptibility of pancreas development to variations in GATA gene dosage in humans versus mice, as evidenced by the absence of noteworthy pancreatic phenotypes in heterozygous GATA4 or GATA6 mice.^37^ Allen et al detected GATA6 mutations in 15 out of 27 individuals afflicted with pancreatic agenesis, constituting 56% of all cases. This finding underscores the fundamental and distinct function of GATA6 in the developmental processes of the human pancreas.^27^

## GATA6 in pancreatic inflammation

Kwei et al conducted an array-based genomic analysis on xenografts of pancreatobiliary cancers, revealing elevated GATA6 expression in cases of pancreatitis, a recognized predisposing factor for pancreatic cancer development.^39^ This observation implies a potential mechanistic association.^40^

An early occurrence during inflammation in the gastrointestinal tract involves the elevation of secretory peptides associated with the trefoil factor family (TFF), which facilitate cell migration and provide mucosal protection and healing. Al-azzeh et al utilized reverse transcription-PCR to illustrate GATA6 expression across various tumor cell lines originating from the pancreas, stomach, and intestines. Their findings strongly indicate that GATA6 functions as a transcriptional activator for TFF1 and TFF2, while not influencing TFF3 in gastric and intestinal cell lines. Intriguingly, GATA6 co-transfection did not exhibit any influence on either TFF1 or TFF2 reporter expression within the three pancreatic cell lines examined (CaPan-2, IMIM-PC1, IMIM-PC2).^41^ The precise rationale behind GATA6's seemingly tissue-specific activation of TFF reporter genes in gastric and intestinal cell lines, coupled with its absence of activation in pancreatic cell lines, remains undisclosed. It is plausible that GATA6 might interact with other factors specific to cells or tissues (present in the stomach and intestine but not in other tissues like the pancreas) to mediate the transcriptional activation of TFFs, as has been shown for GATA1.^42^

## GATA6 in pancreatic cancer pathology

GATA6 emerges as a new addition to the expanding repertoire of cancer-associated genes that hold pivotal roles in regular human development yet assume pathogenic functions in cancer due to deviant expression patterns. In a study conducted by Kwei et al, genomic profiling techniques were employed on a collection of pancreatic and distal bile duct cancers propagated as xenografts within nude mice. In this context, GATA6 was identified and comprehensively characterized as a newfound candidate lineage-specific oncogene, undergoing amplification specifically within pancreatic cancer.^39^ The outcomes of this investigation highlight that the amplification of GATA6 (*i.e.*, the presence of additional gene copies) and its abnormal overexpression significantly contribute to the initiation of pancreatic cancer. Additionally, it is implied that mechanisms beyond gene amplification may likely account for the elevation of GATA6 expression in a considerable subset of cases.

**Figure 2:**
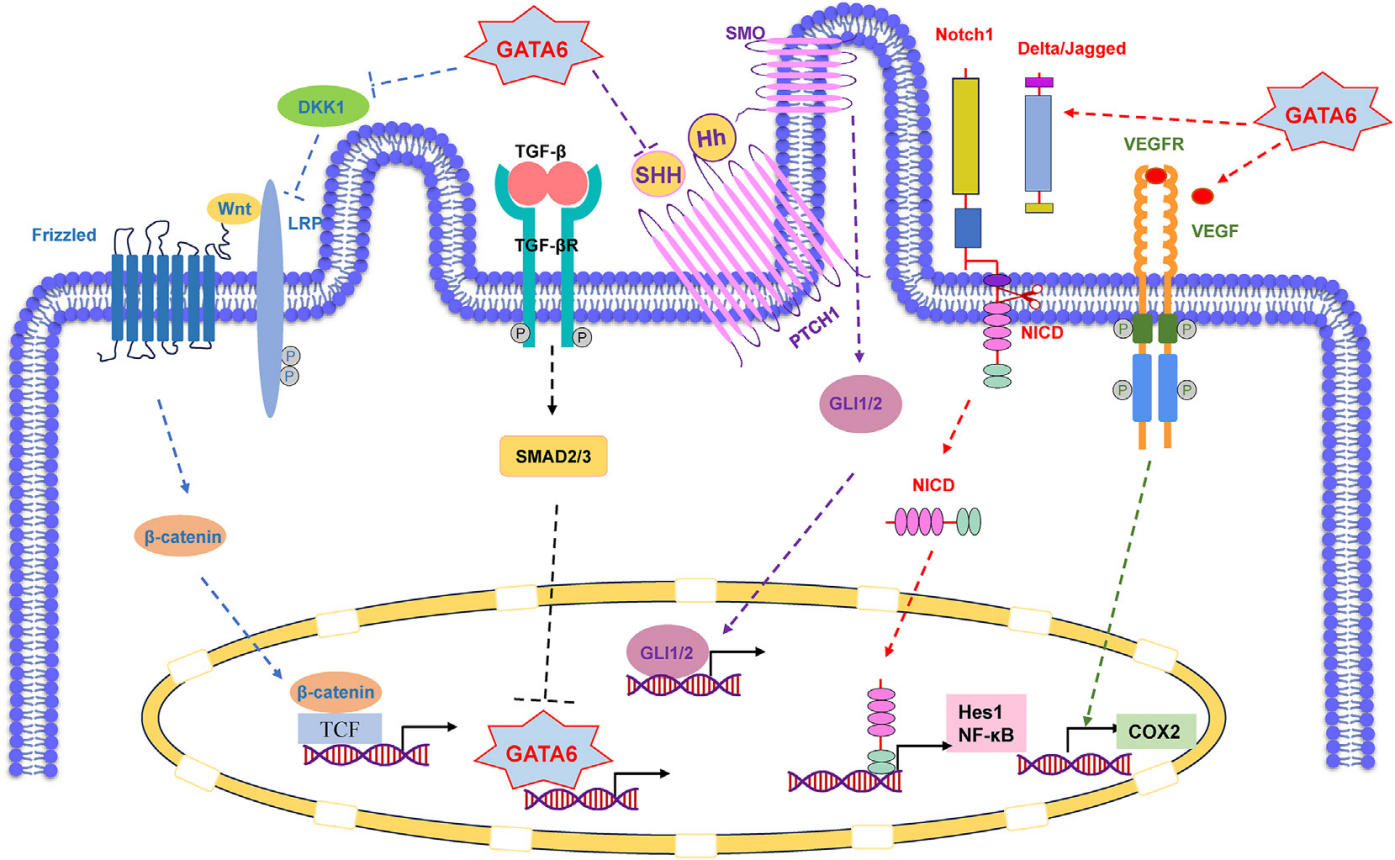
Major signaling pathways associated with GATA6 and pancreas. Wnt antagonist dickkopf1 (DKK1) is a target gene of GATA6, and GATA6 can promote the development and carcinogenesis of the pancreas by activating the Wnt pathway via repressing of DKK1. GATA6 can also regulate the pancreatic endoderm specification through hedgehog signaling by inhibiting sonic hedgehog (Shh), in company with GATA4. GATA6 is the downstream target of the transforming growth factor-β (TGF-β) pathway, and the expression changes of GATA6 can affect the differentiation of pancreatic cells and the self-renewal of pancreas progenitor. In addition, GATA6 can affect the maintenance of intestinal epithelial and goblet cell differentiation by regulating Notch signaling. GATA6 regulates the lymphatic dissemination of bladder cancer through vascular endothelial growth factor receptor (VEGFR) signaling, which may also be associated with the initiation and progression of pancreatic cancer.

Considering its association with developmental processes and cellular specification, the emergence of an oncogenic role for GATA6 might appear unexpected. Notably, GATA6 has been designated as a tumor suppressor gene in alternative cellular contexts,^43^^,^^44^ with instances of inactivating mutations being identified in human malignant astrocytomas.^43^ However, it is worth noting that similar occurrences have been witnessed with other cell lineage-specific transcription factors, such as microphthalmia-associated transcription factor (MITF) in melanoma,^45^ androgen receptor (AR) in hormone-independent prostate cancer,^46^ estrogen receptor alpha (ESR1) in breast cancer,^47^ and more recently, NK2 homeobox 1 (NKX2-1, also known as thyroid transcription factor-1/TITF-1) in lung cancer.^48^ The aberrant expression of these transcriptional regulators, typically involved in lineage proliferation or survival, may be essential for the maintenance and progression of tumors within specific cellular and genetic contexts, indicating a state of “lineage-dependency”.^49^ In a broader sense, the disarrayed expression of transcription factors crucial for regular development corresponds to the concept of “oncology recapitulating ontogeny”.^50^ While research has indicated the predominant amplification of GATA6 in pancreaticobiliary cancers, it is worth noting that GATA6 expression extends beyond the developmental pancreas. Consequently, it remains an open question whether GATA6 might assume an oncogenic role in other cell lineages. The abnormal expression of GATA6 may also affect the pancreas development and carcinogenesis via Wnt, Notch, hedgehog, transforming growth factor-β (TGF-β), and vascular endothelial growth factor receptor (VEGFR) signaling pathways (Fig. 2), which play crucial roles in the initiation and progression of pancreatic cancer.

Another characteristic associated with lineage-specific oncogenes is their tendency to display oncogenic activity that is notably contingent upon the specific cellular and genetic context. For instance, the expression of MITF exhibits growth-restrictive effects in regular human melanocytes.^51^ However, when set within the backdrop of BRAF activation (concurrently accompanied by inactivation of TP53 and RB1 pathways), MITF contributes to both growth factor stimulation and anchorage-independent growth.^45^ Similarly, TITF-1 demonstrates growth-suppressive tendencies in immortalized human lung epithelial cells,^52^ yet propels cell proliferation and viability when amplified in the context of lung cancers.^48^^,^^53^ Consistent with these findings, the expression of GATA6 was observed to exert detrimental effects on the fitness of immortalized human pancreatic ductal epithelial cells, as well as in a PDA cell line (PL45) characterized by KRAS activation but lacking 18q11.2 gain. Further investigations are warranted to elucidate the specific genetic context governing the oncogenic role of GATA6.

### In pancreatic premalignancy

The prevalent majority of PDA cases are posited to originate from minuscule precursor lesions termed pancreatic intraepithelial neoplasia (PanIN). Conversely, a minority of PDA instances emerge in conjunction with cystic lesions present within the pancreas, with intraductal papillary mucinous neoplasms, and mucinous cystic neoplasms constituting the most prevalent forms.^54^^,^^55^ In their study, Fu et al undertook immunolabeling of the GATA6 protein in both normal ducts and samples representing varying stages of PanINs, as well as samples of infiltrating pancreatic cancer. The outcomes revealed that while the labeling of early-stage PanINs exhibited no significant disparities when contrasted with normal duct epithelium or each other, a striking divergence emerged when comparing normal epithelium with samples of PanIN3 or infiltrating cancer. In these stages of the disease, the nuclear labeling of GATA6 displayed notably heightened intensity and was present in a substantial majority of cells, often surpassing 80%. Notably, no discrepancies were identified in the comparison between PanIN3 labeling and infiltrating carcinoma, suggesting that the elevation of GATA6 occurs during the later stages of carcinogenesis, but preceding the development of infiltrating carcinoma.^56^

### In PDA

GATA6 exhibits anomalous expression patterns in PDA. Its amplification, resulting in an increase in gene copy numbers, consequently contributes to the advancement of PDA. Additionally, GATA6 expression is likely to be heightened through mechanisms beyond gene amplification within a notable subset of pancreatobiliary cancer cases.^39^ Distinctive patterns of hydroxymethylation, leading to decreased expression, are discernible in the initial stages of PDA.^57^ Suppression of GATA6 in PDA cells is correlated with diminished proliferation.^39^ GATA6 intervenes in preventing dedifferentiation and the acquisition of metastatic traits in PDA cells. The abatement of GATA6 results in an augmented dissemination of tumor cells.^58^

### In metastasis

Epithelial-mesenchymal transition constitutes a pivotal mechanism driving the invasion and metastasis processes in PDA. GATA6 plays a role in promoting epithelial characteristics and suppressing epithelial–mesenchymal transition within the context of PDA. This role is executed through a distinctive and unparalleled mechanism, entailing the activation of epithelial genes coupled with the concurrent suppression of mesenchymal genes. Furthermore, the influence exerted by GATA6 is twofold; it operates directly by governing the behavior of both epithelial and mesenchymal genes and indirectly by orchestrating the activities of pro-epithelial and pro-mesenchymal transcription factors. Evidently, GATA6 holds the distinction of being the foremost epithelial–mesenchymal transition regulator to possess such attributes.^58^ In mice, GATA6 has also been observed to stifle the phenomenon termed “epithelial-to-epithelial transition”.^59^

### In pancreatic cancer subtypes

A previous study has revealed that the amplification of GATA6 and the subsequent outcome of heightened expression make substantial contributions (although at moderate magnitudes) to the emergence of oncogenic traits, including cellular proliferation, progression through the cell cycle, and the formation of colonies, within cells of pancreatic cancer.^39^ This alignment is consistent with the classification of PDA subtypes.

Recently, there has been progress in utilizing the GATA6 level for the classification of these molecular subtypes. The identification of the basal-like subtype holds vital importance in rationalizing treatments tailored to specific subtypes. O'Kane et al demonstrated that patients with tumors exhibiting a modified “basal-like” phenotype showcase diminished GATA6 RNA expression, while those manifesting the “classical” phenotype are accurately distinguished through elevated GATA6 expression and positive GATA6 staining via *in situ* hybridization.^60^ Another study stemming from the COMPASS trial discovered that the measurement of GATA6 expression in tumors using RNA *in situ* hybridization serves as a robust surrogate biomarker for distinguishment between classical and basal-like subtypes of PDA.^61^ GATA6 undertakes the repression of a basal-like transcriptional program in PDA, and an overexpression signature of GATA6 is enriched in the classical PDA subtype.^18^ The attenuation of canonical differentiation in PDA is associated with reduced GATA6 expression.^20^ A plausible mechanistic explanation involves a causal role of GATA6 in repressing the basal-like program in PDA.^58^

### GATA6 in pancreatic cancer detection and therapy

Distinctive patterns of hydroxymethylation are observed across a multitude of genes, with the most notable effects being evident in genes associated with the development or function of the pancreas, which include GATA6. Modifications in 5-hydroxymethylcytosine facilitate the categorization of PDA, even in the initial phases of the disease.^57^ Individuals harboring tumors displaying an altered “basal-like” phenotype can be discerned through the manifestation of reduced GATA6 expression as determined by RNA sequencing or *in situ* hybridization.^60^ The immunohistochemical analysis of GATA6 can additionally function as a solitary biomarker for prognosticating clinical outcomes in advanced PDA. Remarkably, the utilization of digital support can significantly enhance the immunohistochemical evaluation of GATA6 by pathologists.^62^ The practical utility of RNA sequencing and tumor enrichment through laser capture microdissection is presently constrained due to factors such as tissue acquisition, expenses, and the duration required for reporting. Consequently, employing immunohistochemical analysis to detect GATA6 during diagnosis is an appealing alternative approach for transcriptomic classifiers.

The depletion of GATA6 induces the activation of the epidermal growth factor receptor (EGFR) pathway within PDA cells and in murine PDA models,^59^ indicating a potential predictive or causal function of GATA6 in influencing the response to treatment in patients. Neoptolemos et al analyzed the specimens obtained from patients enrolled in ESPAC-3, a randomized adjuvant trial contrasting 5-fluorouracil/leucovorin and gemcitabine as treatments.^63^ Their findings demonstrate that individuals with GATA6-deficient tumors do not experience advantages from adjuvant 5-fluorouracil/leucovorin therapy and exhibit notably diminished survival rates compared with patients with GATA6-enriched tumors who underwent similar treatment. In contrast, the expression of GATA6 did not exhibit a correlation with the response to gemcitabine treatment.^58^ O'Kane et al proposed that tumors with a basal-like phenotype, or those characterized by diminished GATA6 RNA expression, manifest heightened resistance to modified FOLFIRINOX (mFFX) therapy.^60^ The potential influence of GATA6 expression on the response to oxaliplatin treatment remains to be examined; notably, a prior study involving colorectal cancer has indicated that enhancing GATA6 protein levels might augment the resistance of colorectal cancer stem cells to oxaliplatin.^64^ Collectively, these findings indicate that GATA6 could serve as a predictive indicator for assessing treatment responsiveness and subsequently facilitating patient stratification.

Recognizing the pivotal nature of identifying the basal-like subtype and acknowledging GATA6's limitations, the endeavor to discover supplementary biomarkers for potential combinations appears more practical for clinical practitioners. Keratin 5 emerges as a favorable basal-like biomarker, offering optimal prognostic insights after GATA6 expression, and displaying a marked alignment with GATA6 staining patterns and RNA expression levels. The amalgamation of keratin 5 and GATA6 assessments through consecutive sections and dual immunostaining techniques can successfully unveil the coexistence of basal-like and classical components within a subset of PDA cases.^60^ Furthermore, the simultaneous evaluation of human equilibrative nucleoside transporter 1 (hENT-1) and GATA6 expression might reveal an augmented predictive capacity.^58^

Given that GATA6 is present in typical adult tissues such as the endocrine pancreas, lung, liver, and heart,^34^ its potential as a therapeutic target remains limited. Nevertheless, forthcoming research endeavors will likely establish the precise transcriptional effectors and pathways that underlie GATA6's oncogenic role, potentially identifying significant molecular targets for future exploration.

## Conclusion and future directions

The presence of the development-associated transcription factor GATA6 could potentially hold crucial and previously unacknowledged significance in the initiation of pancreatic carcinogenesis. This comprehensive review has offered enlightening insights into the indispensable roles GATA6 assumes in pancreas development, physiological processes, inflammation, and the intricate landscape of pancreatic cancer pathology. Further explorations into the functional aspects of GATA6 in the context of PDA development and advancement are merited and could contribute to refining the management strategies for this dead disease.

## Funding

This work was supported by the Guangzhou Ruiqian Biotech Company (No. 20230330 to T.J.), 10.13039/501100001809National Natural Science Foundation of China (No. 82072632), Guangzhou Municipality Bureau of Science and Technology (China) (No. 202102010033 to K.X.), and the 10.13039/501100003453Natural Science Foundation of Guangdong Province, China (No. 2022A1515012585 to K.X.).

## Author contributions

M.M., J.A., and T.J. researched the data for the article, wrote the article, and reviewed and/or edited the manuscript before its submission. K.X. contributed to the discussions and editing of the content.

## Conflict of interests

All authors declared no conflict of interests.

## References

[1] Klöppel G., Lüttges J. (2001). WHO-classification 2000: exocrine pancreatic tumors. Verh Dtsch Ges Pathol.

[2] Kardon D.E., Thompson L.D., Przygodzki R.M., Heffess C.S. (2001). Adenosquamous carcinoma of the pancreas: a clinicopathologic series of 25 cases. Mod Pathol.

[3] Liaquat M.T., Kasi A. (July 3, 2023). StatPearls.

[4] Siegel R.L., Miller K.D., Wagle N.S., Jemal A. (2023). Cancer statistics, 2023. CA Cancer J Clin.

[5] Rahib L., Smith B.D., Aizenberg R., Rosenzweig A.B., Fleshman J.M., Matrisian L.M. (2014). Projecting cancer incidence and deaths to 2030: the unexpected burden of thyroid, liver, and pancreas cancers in the United States. Cancer Res.

[6] Biankin A.V., Waddell N., Kassahn K.S. (2012). Pancreatic cancer genomes reveal aberrations in axon guidance pathway genes. Nature.

[7] Waddell N., Pajic M., Patch A.M. (2015). Whole genomes redefine the mutational landscape of pancreatic cancer. Nature.

[8] Jones S., Zhang X., Parsons D.W. (2008). Core signaling pathways in human pancreatic cancers revealed by global genomic analyses. Science.

[9] Swords D.S., Firpo M.A., Scaife C.L., Mulvihill S.J. (2016). Biomarkers in pancreatic adenocarcinoma: current perspectives. OncoTargets Ther.

[10] Fonseca A.L., Kirkwood K., Kim M.P., Maitra A., Koay E.J. (2018). Intraductal papillary mucinous neoplasms of the pancreas: current understanding and future directions for stratification of malignancy risk. Pancreas.

[11] Elta G.H., Enestvedt B.K., Sauer B.G., Lennon A.M. (2018). ACG clinical guideline: diagnosis and management of pancreatic cysts. Am J Gastroenterol.

[12] Ballehaninna U.K., Chamberlain R.S. (2012). The clinical utility of serum CA 19-9 in the diagnosis, prognosis and management of pancreatic adenocarcinoma: an evidence based appraisal. J Gastrointest Oncol.

[13] Biankin A.V., Hudson T.J. (2011). Somatic variation and cancer: therapies lost in the mix. Hum Genet.

[14] Collisson E.A., Bailey P., Chang D.K., Biankin A.V. (2019). Molecular subtypes of pancreatic cancer. Nat Rev Gastroenterol Hepatol.

[15] Perou C.M., Sørlie T., Eisen M.B. (2000). Molecular portraits of human breast tumours. Nature.

[16] Alizadeh A.A., Eisen M.B., Davis R.E. (2000). Distinct types of diffuse large B-cell lymphoma identified by gene expression profiling. Nature.

[17] Nicolle R., Blum Y., Marisa L. (2017). Pancreatic adenocarcinoma therapeutic targets revealed by tumor-stroma cross-talk analyses in patient-derived xenografts. Cell Rep.

[18] Moffitt R.A., Marayati R., Flate E.L. (2015). Virtual microdissection identifies distinct tumor- and stroma-specific subtypes of pancreatic ductal adenocarcinoma. Nat Genet.

[19] Hayashi A., Fan J., Chen R. (2020). A unifying paradigm for transcriptional heterogeneity and squamous features in pancreatic ductal adenocarcinoma. Nat Cancer.

[20] Collisson E.A., Sadanandam A., Olson P. (2011). Subtypes of pancreatic ductal adenocarcinoma and their differing responses to therapy. Nat Med.

[21] Bailey P., Chang D.K., Nones K. (2016). Genomic analyses identify molecular subtypes of pancreatic cancer. Nature.

[22] Puleo F., Nicolle R., Blum Y. (2018). Stratification of pancreatic ductal adenocarcinomas based on tumor and microenvironment features. Gastroenterology.

[23] Peng J., Sun B.F., Chen C.Y. (2019). Single-cell RNA-seq highlights intra-tumoral heterogeneity and malignant progression in pancreatic ductal adenocarcinoma. Cell Res.

[24] Dijk F., Veenstra V.L., Soer E.C. (2020). Unsupervised class discovery in pancreatic ductal adenocarcinoma reveals cell-intrinsic mesenchymal features and high concordance between existing classification systems. Sci Rep.

[25] Juiz N., Elkaoutari A., Bigonnet M. (2020). Basal-like and classical cells coexist in pancreatic cancer revealed by single-cell analysis on biopsy-derived pancreatic cancer organoids from the classical subtype. FASEB J.

[26] Maeda M., Ohashi K., Ohashi-Kobayashi A. (2005). Further extension of mammalian GATA-6. Dev Growth Differ.

[27] Allen H.L., Flanagan S.E., Shaw-Smith C. (2011). GATA6 haploinsufficiency causes pancreatic agenesis in humans. Nat Genet.

[28] Bresnick E.H., Lee H.Y., Fujiwara T., Johnson K.D., Keles S. (2010). GATA switches as developmental drivers. J Biol Chem.

[29] Patient R.K., McGhee J.D. (2002). The GATA family (vertebrates and invertebrates). Curr Opin Genet Dev.

[30] Chen H.V., Lorenzini M.H., Lavalle S.N. (2023). Deletion mapping of regulatory elements for GATA3 in T cells reveals a distal enhancer involved in allergic diseases. Am J Hum Genet.

[31] Molkentin J.D. (2000). The zinc finger-containing transcription factors GATA-4, -5, and -6. Ubiquitously expressed regulators of tissue-specific gene expression. J Biol Chem.

[32] Brewer A., Pizzey J. (2006). GATA factors in vertebrate heart development and disease. Expert Rev Mol Med.

[33] Zhao J., Ohsumi T.K., Kung J.T. (2010). Genome-wide identification of polycomb-associated RNAs by RIP-seq. Mol Cell.

[34] Suzuki H., Gabrielson E., Chen W. (2002). A genomic screen for genes upregulated by demethylation and histone deacetylase inhibition in human colorectal cancer. Nat Genet.

[35] Ketola I., Otonkoski T., Pulkkinen M.A. (2004). Transcription factor GATA-6 is expressed in the endocrine and GATA-4 in the exocrine pancreas. Mol Cell Endocrinol.

[36] Decker K., Goldman D.C., Grasch C.L., Sussel L. (2006). Gata6 is an important regulator of mouse pancreas development. Dev Biol.

[37] Xuan S., Borok M.J., Decker K.J. (2012). Pancreas-specific deletion of mouse Gata4 and Gata6 causes pancreatic agenesis. J Clin Investig.

[38] Carrasco M., Delgado I., Soria B., Martín F., Rojas A. (2012). GATA4 and GATA6 control mouse pancreas organogenesis. J Clin Investig.

[39] Kwei K.A., Bashyam M.D., Kao J. (2008). Genomic profiling identifies GATA6 as a candidate oncogene amplified in pancreatobiliary cancer. PLoS Genet.

[40] Algül H., Treiber M., Lesina M., Schmid R.M. (2007). Mechanisms of disease: chronic inflammation and cancer in the pancreas: a potential role for pancreatic stellate cells?. Nat Clin Pract Gastroenterol Hepatol.

[41] Al-azzeh E.D., Fegert P., Blin N., Gött P. (2000). Transcription factor GATA-6 activates expression of gastroprotective trefoil genes TFF_1_ and TFF_2_. Biochim Biophys Acta.

[42] Sakai Y., Nakagawa R., Sato R., Maeda M. (1998). Selection of DNA binding sites for human transcriptional regulator GATA-6. Biochem Biophys Res Commun.

[43] Kamnasaran D., Qian B., Hawkins C., Stanford W.L., Guha A. (2007). GATA6 is an astrocytoma tumor suppressor gene identified by gene trapping of mouse glioma model. Proc Natl Acad Sci U S A.

[44] Capo-chichi C.D., Roland I.H., Vanderveer L. (2003). Anomalous expression of epithelial differentiation-determining GATA factors in ovarian tumorigenesis. Cancer Res.

[45] Garraway L.A., Widlund H.R., Rubin M.A. (2005). Integrative genomic analyses identify MITF as a lineage survival oncogene amplified in malignant melanoma. Nature.

[46] Visakorpi T., Hyytinen E., Koivisto P. (1995). *In vivo* amplification of the androgen receptor gene and progression of human prostate cancer. Nat Genet.

[47] Holst F., Stahl P.R., Ruiz C. (2007). Estrogen receptor alpha (*ESR1*) gene amplification is frequent in breast cancer. Nat Genet.

[48] Kwei K.A., Kim Y.H., Girard L. (2008). Genomic profiling identifies TITF1 as a lineage-specific oncogene amplified in lung cancer. Oncogene.

[49] Garraway L.A., Sellers W.R. (2006). Lineage dependency and lineage-survival oncogenes in human cancer. Nat Rev Cancer.

[50] Lechner J.F., Fugaro J.M., Wong Y., Pass H.I., Harris C.C., Belinsky S.A. (2001). Perspective: cell differentiation theory may advance early detection of and therapy for lung cancer. Radiat Res.

[51] Loercher A.E., Tank E.M.H., Delston R.B., Harbour J.W. (2005). MITF links differentiation with cell cycle arrest in melanocytes by transcriptional activation of INK4A. J Cell Biol.

[52] Tanaka H., Yanagisawa K., Shinjo K. (2007). Lineage-specific dependency of lung adenocarcinomas on the lung development regulator TTF-1. Cancer Res.

[53] Weir B.A., Woo M.S., Getz G. (2007). Characterizing the cancer genome in lung adenocarcinoma. Nature.

[54] Hruban R.H., Takaori K., Klimstra D.S. (2004). An illustrated consensus on the classification of pancreatic intraepithelial neoplasia and intraductal papillary mucinous neoplasms. Am J Surg Pathol.

[55] Basturk O., Hong S.M., Wood L.D. (2015). A revised classification system and recommendations from the Baltimore consensus meeting for neoplastic precursor lesions in the pancreas. Am J Surg Pathol.

[56] Fu B., Luo M., Lakkur S., Lucito R., Iacobuzio-Donahue C.A. (2008). Frequent genomic copy number gain and overexpression of GATA-6 in pancreatic carcinoma. Cancer Biol Ther.

[57] Guler G.D., Ning Y., Ku C.J. (2020). Detection of early stage pancreatic cancer using 5-hydroxymethylcytosine signatures in circulating cell free DNA. Nat Commun.

[58] Martinelli P., Pau E.C.D.S., Cox T. (2017). GATA6 regulates EMT and tumour dissemination, and is a marker of response to adjuvant chemotherapy in pancreatic cancer. Gut.

[59] Martinelli P., Madriles F., Cañamero M. (2016). The acinar regulator Gata6 suppresses KrasG12V-driven pancreatic tumorigenesis in mice. Gut.

[60] O'Kane G.M., Grünwald B.T., Jang G.H. (2020). GATA6 expression distinguishes classical and basal-like subtypes in advanced pancreatic cancer. Clin Cancer Res.

[61] Aung K.L., Fischer S.E., Denroche R.E. (2018). Genomics-driven precision medicine for advanced pancreatic cancer: early results from the COMPASS trial. Clin Cancer Res.

[62] Duan K., Jang G.H., Grant R.C. (2021). The value of GATA6 immunohistochemistry and computer-assisted diagnosis to predict clinical outcome in advanced pancreatic cancer. Sci Rep.

[63] Neoptolemos J.P., Stocken D.D., Bassi C. (2010). Adjuvant chemotherapy with fluorouracil plus folinic acid *vs* gemcitabine following pancreatic cancer resection: a randomized controlled trial. JAMA.

[64] Sun J., Zhou H., Bao X. (2021). lncRNA TUG1 facilitates colorectal cancer stem cell characteristics and chemoresistance by enhancing GATA6 protein stability. Stem Cells Int.

